# The *ARK2N* (*C18ORF25*) Genetic Variant Is Associated with Muscle Fiber Size and Strength Athlete Status

**DOI:** 10.3390/metabo14120684

**Published:** 2024-12-05

**Authors:** Rukiye Çığırtaş, Celal Bulgay, Hasan Hüseyin Kazan, Onur Akman, Goran Sporiš, George John, Rinat A. Yusupov, Rinat I. Sultanov, Andrey V. Zhelankin, Ekaterina A. Semenova, Andrey K. Larin, Nikolay A. Kulemin, Edward V. Generozov, Damir Jurko, Ildus I. Ahmetov

**Affiliations:** 1 Faculty of Sports Sciences, Bingol University, 12000 Bingol, Türkiye; 2 Department of Medical Biology, Gulhane Faculty of Medicine, University of Health Sciences, 06018 Ankara, Türkiye; 3 Faculty of Sports Sciences, Bayburt University, 69000 Bayburt, Türkiye; onurakman@bayburt.edu.tr; 4 Department of General and Applied Kinesiology, Faculty of Kinesiology, Zagreb University, 10110 Zagreb, Croatia; 5 Transform Specialist Medical Centre, Dubai 119190, United Arab Emirates; 6 Department of Physical Culture and Sport, Kazan National Research Technical University named after A.N. Tupolev-KAI, 420111 Kazan, Russia; 7 Department of Molecular Biology and Genetics, Lopukhin Federal Research and Clinical Center of Physical-Chemical Medicine of Federal Medical Biological Agency, 119435 Moscow, Russiagenerozov@rcpcm.org (E.V.G.); 8 Research Institute of Physical Culture and Sport, Volga Region State University of Physical Culture, Sport and Tourism, 420138 Kazan, Russia; 9 Sports Genetics Laboratory, St Petersburg Research Institute of Physical Culture, 191040 St Petersburg, Russia; 10 Laboratory of Genetics of Aging and Longevity, Kazan State Medical University, 420012 Kazan, Russia; 11 Research Institute for Sport and Exercise Sciences, Liverpool John Moores University, Liverpool L3 5AF, UK

**Keywords:** polymorphism, genotype, gene expression, SNP, athlete status, weightlifting, sport, molecular physiology, muscle hypertrophy, skeletal muscle

## Abstract

Background: Data on the genetic factors contributing to inter-individual variability in muscle fiber size are limited. Recent research has demonstrated that mice lacking the Arkadia (RNF111) N-terminal-like PKA signaling regulator 2N (*Ark2n*; also known as *C18orf25*) gene exhibit reduced muscle fiber size, contraction force, and exercise capacity, along with defects in calcium handling within fast-twitch muscle fibers. However, the role of the *ARK2N* gene in human muscle physiology, and particularly in athletic populations, remains poorly understood. The aim of this study was threefold: (a) to compare *ARK2N* gene expression between power and endurance athletes; (b) to analyze the relationship between *ARK2N* gene expression and muscle fiber composition; and (c) to investigate the association between the functional variant of the *ARK2N* gene, muscle fiber size, and sport-related phenotypes. Results: We found that *ARK2N* gene expression was significantly higher in power athletes compared to endurance athletes (*p* = 0.042) and was positively associated with the proportion of oxidative fast-twitch (type IIA) muscle fibers in untrained subjects (*p* = 0.017, adjusted for age and sex). Additionally, we observed that the *ARK2N* rs6507691 T allele, which predicts high *ARK2N* gene expression (*p* = 3.8 × 10^−12^), was associated with a greater cross-sectional area of fast-twitch muscle fibers in strength athletes (*p* = 0.015) and was over-represented in world-class strength athletes (38.6%; OR = 2.2, *p* = 0.023) and wrestlers (33.8%; OR = 1.8, *p* = 0.044) compared to controls (22.0%). Conclusions: In conclusion, *ARK2N* appears to be a gene specific to oxidative fast-twitch myofibers, with its functional variant being associated with muscle fiber size and strength-athlete status.

## 1. Introduction

Strength is a fundamental component of athletic performance, with muscle strength and power being essential in competitive sports requiring high-force outputs, such as weightlifting, sprinting, and jumping. Muscle strength is known to be a highly heritable trait, with heritability estimates for strength-related phenotypes ranging from 30% to 80% [1,2,3]. Genetic contributions to strength are polygenic, involving numerous genetic variants that collectively influence the phenotype [4,5,6,7,8,9,10].

This complex genetic architecture underscores the diversity of biological factors contributing to strength performance, including greater muscle fiber size [11], the prevalence of oxidative fast-twitch (type IIA) muscle fibers [12], shifts in muscle metabolism toward glycolysis [13], increased testosterone levels [14], and neural adaptations that enhance force production capabilities [15].

Research on the genetics of strength and related phenotypes has advanced significantly in recent years, especially with the use of genome-wide approaches [4,5,6,16,17,18]. This research has identified over 40 genetic variants associated with strength-athlete status and weightlifting performance, including those located near or within the *ABHD17C*, *ACE*, *ACTG1*, *ACTN3*, *ADCY3*, *ADPGK*, *AGT*, *ALDH2*, *ANGPT2*, *AR*, *ARPP21*, *BCDIN3D*, *CKM*, *CNTFR*, *CRTAC1*, *DHODH*, *GALNTL6*, *GBE1*, *GBF1*, *GLIS3*, *HIF1A*, *IGF1*, *IL6*, *ITPR1*, *KIF1B*, *LRPPRC*, *MLN*, *MMS22L*, *MTHFR*, *NPIPB6*, *PHACTR1*, *PLEKHB1*, *PPARA*, *PPARG*, *PPARGC1A*, *R3HDM1*, *RASGRF1*, *RMC1*, *SLC39A8*, *TFAP2D*, *ZKSCAN5*, and *ZNF608* genes [7,8,9,19,20,21,22,23,24,25,26,27,28,29,30,31,32,33,34,35,36,37,38,39]. Among these, polymorphisms in the *ACTN3*, *AR*, *LRPPRC*, *MMS22L*, *PHACTR1*, and *PPARG* genes are particularly promising, with each variant being shown to play a role in specific physiological pathways that contribute to muscle strength and performance [40,41].

Overall, the study of genetic contributions to strength provides a foundation for understanding individual variability in athletic performance and offers pathways for personalized approaches in sports training and rehabilitation. As research continues to map the genetic landscape of strength, identifying specific markers and their physiological roles will enhance our understanding of how genetics shape strength-related traits and inform precision-based interventions to maximize human potential in sports and health contexts [9,10].

Recently, a new and promising candidate gene related to strength performance and skeletal muscle hypertrophy has been identified [42]. Specifically, research has demonstrated that mice lacking the *Ark2n* gene (also known as *C18orf25*; chromosome 18 open reading frame 25) exhibit a reduced muscle fiber size, contraction force, and exercise capacity, along with defects in calcium handling within fast-twitch muscle fibers [42,43]. The *ARK2N* gene encodes a protein known as Arkadia (RNF111) N-terminal-like PKA signaling regulator 2N (ARK2N), which is homologous to ring finger protein 111 (RNF111), an E3 ubiquitin ligase. Unlike RNF111, ARK2N lacks the domain necessary for ubiquitin binding and is therefore considered an adaptor or signaling protein without ubiquitination activity [44]. Furthermore, ARK2N has been shown to undergo phosphorylation by AMP-activated protein kinase (AMPK) in humans following acute exercise, which enhances skeletal muscle contractile function ex vivo [43].

Given that elite weightlifters typically exhibit a high proportion of oxidative fast-twitch (type IIA) muscle fibers [12], we hypothesized that strength-related *ARK2N* gene expression is positively correlated with the proportion of type IIA muscle fibers and that functional variants within the *ARK2N* gene are associated with elite weightlifting performance and power-athlete status. This study aimed to (a) compare *ARK2N* gene expression between power and endurance athletes; (b) analyze the relationship between *ARK2N* gene expression and muscle fiber composition; and (c) investigate the association between the functional variant of the *ARK2N* gene, muscle fiber size, and sport-related phenotypes.

## 2. Materials and Methods

### 2.1. Ethical Approval

This study was approved by the Ethics Committee of Bingol University (reference 23/20; approval date: 9 November 2023) and the Ethics Committees of the Federal Research and Clinical Center of Physical–Chemical Medicine of the Federal Medical and Biological Agency of Russia (reference 2017/04; approval date: 4 July 2017). Written informed consent was obtained from each participant before the start of this study, which complied with the Declaration of Helsinki and ethical standards for sport and exercise science research. 

### 2.2. Study Participants

#### 2.2.1. The Turkish Cohorts

The study included 60 Turkish track and field athletes, consisting of 31 power athletes (11 females and 20 males: 100–400 m runners [*n* = 9], jumpers [*n* = 3], and throwers [*n* = 19]; age 26.6 (3.0) years; sport experience 7.4 (3.6) years) and 29 endurance athletes (10 females and 19 males: 3000 m [*n* = 12], 5000 m [*n* = 5], 10,000 m [*n* = 4], and marathon runners [*n* = 8]; age 27.5 (4.2) years; sport experience 11.5 (5.1) years). All athletes were affiliated with the Turkish Athletics Federation and were ranked within the top ten nationally in their respective disciplines. The control group included 20 healthy, unrelated Turkish individuals without competitive sports experience. Allele frequencies of the *ARK2N* rs6507691 polymorphism were verified by comparison with data from 557 healthy participants in the Turkish Genome Project (https://tgd.tuseb.gov.tr/en/; accessed on 5 August 2024). All athletes and controls were of Caucasian ancestry, and none had tested positive for doping.

#### 2.2.2. The Russian Cohorts

The gene-expression study included 10 sub-elite power athletes (four powerlifters, four weightlifters, one decathlete, and one taekwondo athlete; mean age 30.1 ± 7.4 years; height 178.2 ± 6.7 cm; body mass 85.6 ± 12.4 kg) and 13 sub-elite endurance athletes (nine long-distance runners, three triathletes, and one cross-country skier; mean age 34.2 (10.0) years; height 182.8 (6.8) cm; body mass 76.0 (9.9) kg), all of European descent (Russians), as previously described [45]. The muscle-fiber-size study included 24 sub-elite strength athletes (17 powerlifters, 7 weightlifters; mean age 30.0 (5.3) years; height 178.9 (6.3) cm; body mass 90.9 (11.3) kg) of European descent. Additionally, a case-control study involved 90 weightlifters (55 males, mean age 27.7 (5.4) years; 35 females, mean age 26.8 (3.3) years), 125 wrestlers (87 males, mean age 25.7 (4.2) years; 38 females, mean age 29.2 (3.5) years; 38 Greco-Roman wrestlers, 33 freestyle wrestlers, 26 sambo wrestlers, 17 judo wrestlers, and 11 belt wrestlers), and 182 controls (138 males, 44 females; mean age 44.9 (4.2) years). Among the 215 athletes (strength athletes and wrestlers), 56 were world class (Olympic/world/European Championship medalists: 22 weightlifters and 34 wrestlers), while 159 were elite (international level, non-medalists). None of the athletes had tested positive for doping.

#### 2.2.3. The FUSION Cohort

The gene-expression study (vastus lateralis) included 291 sedentary individuals of European descent from the FUSION study [46], comprising 166 men (mean age 59.5 (8.1) years; height 176.7 (6.7) cm; body mass 87.3 (15.1) kg) and 125 women (mean age 60.3 (8.1) years; height 162.8 (5.6) cm; body mass 71.8 (9.8) kg).

### 2.3. Performance Analysis

To analyze the performance levels of the Turkish athletes, their personal bests were evaluated using the World Athletics (formerly IAAF) scoring system, as previously described [17].

### 2.4. Genotyping

Genotyping of the rs6507691 polymorphism in the Turkish participants was performed using either in-house (laboratory-developed) real-time PCR for the athletes or SNP array analysis for the other participants, using genomic DNA isolated from peripheral blood. DNA was isolated using the DNeasy Blood and Tissue Kit (Qiagen, Hilden, Germany) according to the manufacturer’s protocol. Quantification of the isolated DNA was carried out with a NanoDrop1000 spectrophotometer (Thermo Scientific, Waltham, MA, USA). Real-time PCR was conducted using a TaqMan probe-based strategy. The primer–probe sets (forward primer: 5′ GCCCAAAAGTCGTGTCCCTT 3′; reverse primer: 5′ ATTAGCTGGCGTAGTGGTGG 3′; Probe-1: 5′ [FAM] CCTCTGCCTCCCAGGTTCAAGCG 3′; Probe-2: 5′ [HEX] CCTCTGCCTCCCAGGCTCAAGCG 3′; Probe-3: 5′ [HEX] CCTCTGCCTCCCAGGGTCAAGCG 3′) were designed using the Primer-BLAST tool (https://www.ncbi.nlm.nih.gov/tools/primer-blast/; accessed on 5 August 2024). The PCR was then performed with the Takyon ROX Probe 2X MasterMix dTTP (Eurogentec, Seraing, Belgium). Each PCR mix consisted of 10 μL of the probe mastermix, 1 μL of 10 μM forward and reverse primers, 0.5 μL of 10 μM Probe-1 and Probe-2 or Probe-3, and 30 ng of genomic DNA. The reactions were conducted on a BioRad CFX 96 instrument (BioRad Inc., Hercules, CA, USA) with the following thermal profile: 95 °C for 5 min for initial denaturation, followed by 40 cycles of 95 °C for 30 s (denaturation), and 60 °C for 30 s (annealing, extension, and signal acquisition on FAM and HEX channels). Negative controls and samples with known rs6507691 genotypes were included. Genotypes of participants were manually determined based on amplification curves. For the Russian athletes and control subjects, molecular genetic analysis was performed with DNA obtained from leukocytes (4 mL of venous blood). DNA extraction and purification were conducted using a commercial kit according to the manufacturer’s instructions (Technoclon, Moscow, Russia). Genotyping of rs6507691 was performed using microarray technology (Illumina, San Diego, CA, USA) with HumanOmni1-Quad and HumanOmniExpress BeadChips (Illumina), as previously described [11].

### 2.5. Gene-Expression Analysis

Transcriptomic analysis was performed as previously described [18,45]. Briefly, to minimize confounding factors related to muscle recovery and prior activity, participants (*n* = 23) were instructed to refrain from training for at least 24 h before the biopsy. This rest period was implemented to ensure the gene-expression profiles reflected a baseline, resting state, without the acute effects of exercise. RNA was isolated from muscle tissue using the RNeasy Mini Fibrous Tissue Kit (Qiagen, Hilden, Germany). RNA concentration was measured using the Qubit spectrophotometer (Thermo Fisher Scientific, Waltham, MA, USA). RNA quality was assessed using the BioAnalyzer electrophoresis system and BioAnalyzer RNA Nano assay (Agilent Technologies, Santa Clara, CA, USA). The RNA integrity number (RIN) was calculated for each RNA sample. Only RNA samples with RIN > 7 were included in the study. Samples were stored at −80 °C until sequencing libraries were prepared. Total RNA samples were treated with DNAse I using the Turbo DNA-free Kit (Thermo Fisher Scientific) according to the kit guidelines. Libraries for RNA sequencing were prepared using the NEBNext Ultra II Directional RNA Library Prep Kit for Illumina with the NEBNext rRNA Depletion Module (New England Biolabs, Ipswich, MA, USA). RNA libraries were sequenced on the HiSeq system (Illumina) for 250 cycles. Gene-level expression abundances were calculated using the tximport Bioconductor package [47], with *ARK2N* gene expression presented in transcripts per kilobase million (TPM). Transcriptome analyses of muscle samples from the FUSION cohorts were described by Taylor et al. [46].

### 2.6. Evaluation of Muscle Fiber Composition

The muscle fiber composition of the vastus lateralis in strength athletes was assessed using immunohistochemistry, as previously described [11]. Briefly, samples from the left vastus lateralis were collected using the modified Bergström needle technique. Prior to analysis, samples were frozen in liquid nitrogen and stored at −80 °C. Serial cross-sections (7 μm) were obtained from frozen samples using an ultratom (Leica Microsystems, Wetzlar, Germany). Sections were thaw-mounted on Polysine glass slides, maintained at room temperature (RT) for 15 min, and incubated in PBS (3 × 5 min). The sections were then incubated at RT in primary antibodies against slow or fast isoforms of the myosin heavy chains (M8421, 1:5000; M4276; 1:600, respectively; Sigma–Aldrich, St. Louis, MO, USA) for 1 h and incubated in PBS (3 × 5 min). Next, the sections were incubated at RT in secondary antibodies conjugated with FITC (F0257; 1:100; Sigma–Aldrich) for 1 h. The antibodies were removed, and the sections washed in PBS (3 × 5 min), placed in mounting media and covered with a cover slip. Images were captured by fluorescent microscope (Eclipse Ti-U, Nikon, Tokyo, Japan). Fibers stained in serial sections with antibodies against slow and fast isoforms were considered to be hybrid fibers. Muscle fiber composition in the vastus lateralis of the FUSION cohort was estimated based on the expression of *MYH1*, *MYH2*, and *MYH7* genes, as previously described [46].

### 2.7. Statistical Analyses

Statistical analyses were performed using SPSS software version 29.0. Allelic and genotypic frequencies were assessed for the Hardy–Weinberg equilibrium (HWE) and evaluated using the chi-square (χ^2^) or Fisher’s exact test. Sample size and power calculations were performed using G*Power (v3.1) with the chi-squared test for proportions to ensure sufficient power to detect a difference in allelic frequencies between athletes and controls. Associations with alleles or genotypes were determined using SNPStats software v1 [48] with co-dominant, dominant, recessive, and over-dominant models. The SNPStats results were further validated using one-way ANCOVA with sex and sports experience as categorical covariates. A normality test was used to assess the linearity of the data. Mean differences between groups were analyzed using Student’s unpaired *t*-test. Relationships between gene expression and muscle-related traits were assessed using regression analysis adjusted for covariates (age and sex). Scatter plots were generated based on Pearson correlation coefficients. A Pearson correlation was used to calculate the correlation coefficient (*r*) and the coefficient of determination (*R^2^*). All data are presented as mean (SD), with *p*-values < 0.05 considered statistically significant.

## 3. Results

### 3.1. Gene-Expression Studies

**Figure 1:**
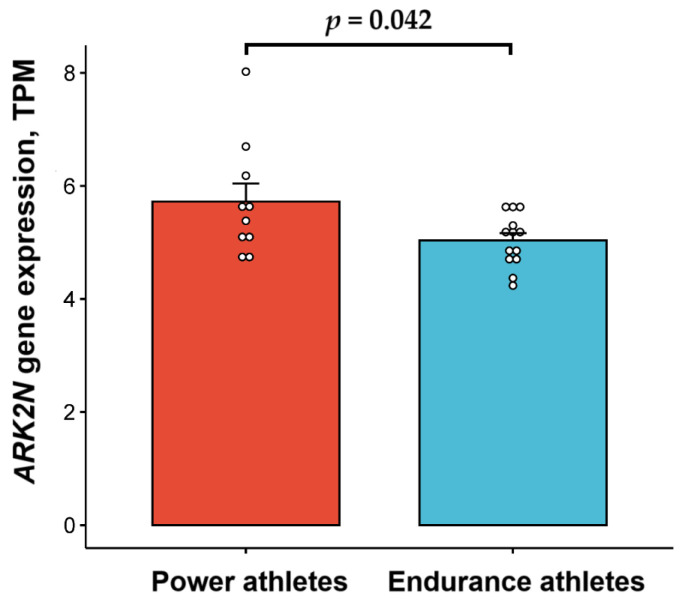
Comparison of Arkadia (RNF111) N-terminal-like PKA signaling regulator 2N (*ARK2N*) gene expression between power and endurance athletes.

*ARK2N* gene expression was significantly higher (5.7 (1.0) vs. 5.0 (0.5) TPM, *p* = 0.042) in the vasus lateralis muscle of power athletes (*n* = 10) compared to endurance athletes (*n* = 13) (Figure 1).

**Figure 2:**
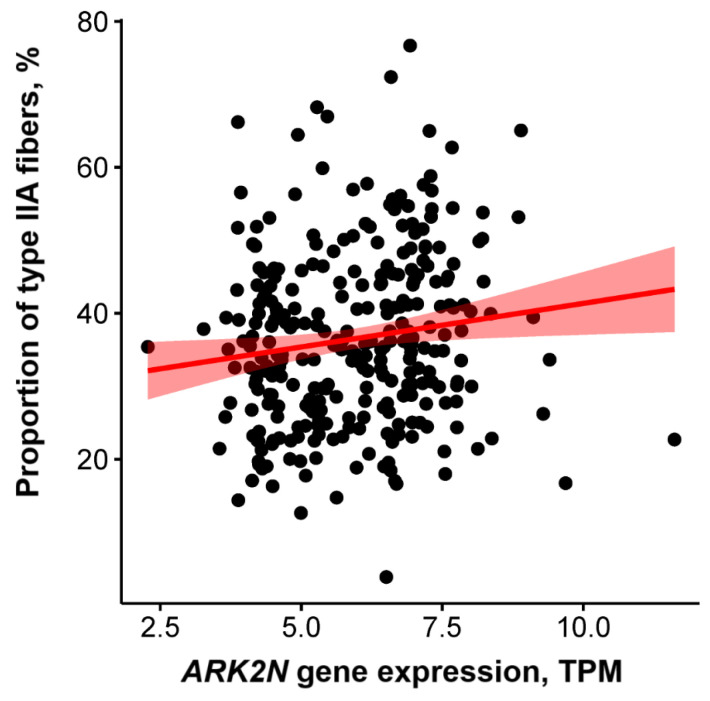
Relationship between Arkadia (RNF111) N-terminal-like PKA signaling regulator 2N (*ARK2N*) gene expression and the proportion of type IIA muscle fibers in the FUSION cohort (*p* = 0.017; adjusted for age and sex).

In the FUSION cohort (*n* = 291), males and females had significantly different proportions of type IIA muscle fibers (33.0% vs. 39.2%, *p* < 0.0001); therefore, we adjusted our results for sex and age. Accordingly, *ARK2N* gene expression was positively associated with the proportion of oxidative fast-twitch (type IIA) muscle fibers in untrained subjects (*p* = 0.017, adjusted for age and sex; females: *p* = 0.029, r = 0.2, *R^2^* = 4%; males: *p* = 0.124, *r* = 0.12, *R^2^* = 1.4%) (Figure 2). This association remained significant (*p* = 0.019) after adjusting for body mass index and smoking status as additional covariates. No significant relationship was observed between *ARK2N* gene expression and the proportion of glycolytic fast-twitch (type IIX) muscle fibers (*p* = 0.654) or slow-twitch (type I) muscle fibers (*p* = 0.242).

### 3.2. Case-Control and Genotype–Phenotype Studies

According to the GTEx portal (https://gtexportal.org/; accessed on 9 November 2024), the *ARK2N* gene includes several functional variants. Among these, the *ARK2N* rs6507691 C/T polymorphism is one of the most significant, with the T allele associated with higher *ARK2N* gene expression (*p* = 3.8 × 10^−12^). We therefore analyzed this polymorphism in the athlete cohorts and control groups.

Genotype distributions and allele frequencies for the *ARK2N* rs6507691 polymorphism in the Turkish and Russian cohorts are presented in Table 1.

**Figure 3:**
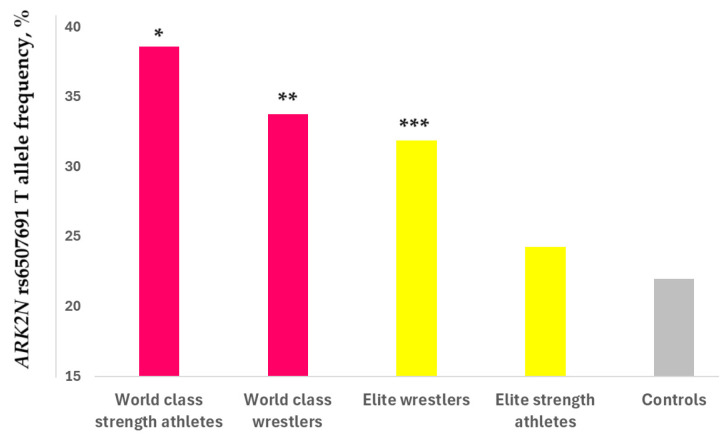
Arkadia (RNF111) N-terminal-like PKA signaling regulator 2N (*ARK2N*) rs6507691 T allele frequency (%) in Russian athletic cohorts (22 world-class strength athletes, 34 world-class wrestlers, 91 elite wrestlers, 68 elite strength athletes) and controls (*n* = 182). * *p* = 0.023, ** *p* = 0.044, and *** *p* = 0.016 indicate statistically significant differences between athletic cohorts and controls.

All genotype distributions were in the Hardy–Weinberg equilibrium (*p* > 0.05). No differences in genotype frequency were observed between Turkish power or endurance athletes and Turkish control groups (*p* > 0.05). However, the *ARK2N* rs6507691 T allele was significantly overrepresented in world-class strength athletes (38.6%; OR = 2.2, *p* = 0.023) and world-class wrestlers (33.8%; OR = 1.8, *p* = 0.044) compared to controls (22.0%) (Figure 3). Additionally, the frequency of the *ARK2N* rs6507691 T allele was significantly higher in elite wrestlers (31.9%, *p* = 0.016) and in the combined Russian athlete group (strength athletes + wrestlers; 30.5%, *p* = 0.0078) (Table 1).

No significant association was found between the *ARK2N* rs6507691 polymorphism and competitive performance in Turkish power and endurance athletes (see Supplementary Tables S1–S3).

### 3.3. Muscle-Fiber-Size Study

**Figure 4:**
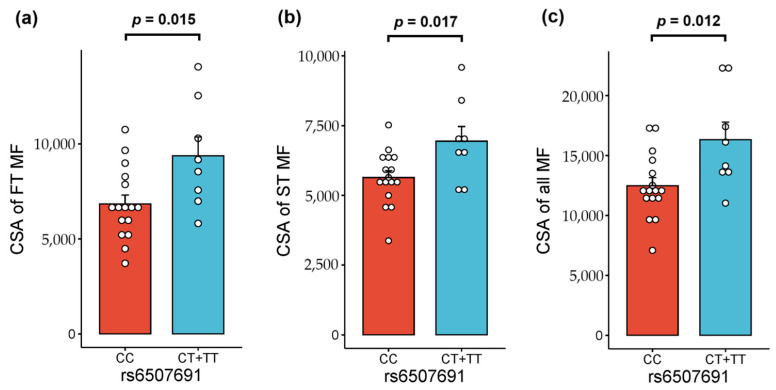
Comparison of cross-sectional area of (**a**) fast-twitch, (**b**) slow-twitch, and (**c**) combined muscle fibers between strength athletes with different Arkadia (RNF111) N-terminal-like PKA signaling regulator 2N (*ARK2N*) rs6507691 genotypes (*n* = 24). Abbreviations: CSA, cross-sectional area; FT MF, fast-twitch muscle fibers; ST MF, slow-twitch muscle fibers; all MF, combined fast-twitch + slow-twitch muscle fibers.

Among the strength athletes (*n* = 24), carriers of the *ARK2N* rs6507691 T allele exhibited a significantly greater cross-sectional area (CSA) of fast-twitch (9366 (2806) µm^2^ vs. 6831 (1884) µm^2^; *p* = 0.015) and slow-twitch (6942 (1490) µm^2^ vs. 5638 (972) µm^2^; *p* = 0.017) muscle fibers, as well as the combined CSA of both muscle fiber types (16,308 (4141) µm^2^ vs. 12,469 (2699) µm^2^; *p* = 0.012) compared to CC homozygotes (Figure 4).

## 4. Discussion

This study is the first to demonstrate that the *ARK2N* (*C18ORF25*) genetic variant is associated with a predisposition toward strength-oriented sports, including weightlifting, powerlifting, and wrestling. Our findings also provide new insights into the role of the *ARK2N* gene in muscle fiber composition and size. The higher expression of *ARK2N* observed in the vastus lateralis of power athletes supports the hypothesis that *ARK2N* plays a specific role in muscle adaptation for power- and strength-related activities. This is further supported by its positive association with the proportion of oxidative fast-twitch (type IIA) muscle fibers, which are abundant in elite weightlifters [12]. These findings align with previous research demonstrating that *ARK2N*, particularly through its AMPK-mediated phosphorylation at S67, is crucial in muscle contractile function and calcium handling [42,43].

The analysis of *ARK2N* rs6507691 further underscores the gene’s relevance to muscle phenotypes associated with strength and power. The rs6507691 C/T polymorphism, located in an intron of the *ARK2N* gene, is considered as an expression quantitative trait locus (eQTL), or genetic locus that explain variations in mRNA expression levels. The T allele, associated with increased *ARK2N* expression, was significantly overrepresented among highly elite strength athletes and wrestlers, suggesting a potential genetic advantage for muscle performance in strength-related sports. Our observation of a greater cross-sectional area (CSA) in both fast-twitch and slow-twitch muscle fibers among T allele carriers aligns with this, indicating that the T allele may contribute to muscle hypertrophy, a beneficial trait for strength sports [49,50]. This effect may be linked to ARK2N’s role in efficient calcium handling, which promotes muscle hypertrophy through the activation of the mTOR pathway, a key regulator of muscle protein synthesis [51]. However, the exact mechanisms underlying the association between the *ARK2N* T allele and the increased CSA of muscle fibers remain to be explored in future studies.

*ARK2N*, also known as *C18ORF25*, has emerged as a novel and critical regulator of skeletal muscle function, particularly regarding exercise physiology and muscle strength. Initially identified through phosphoproteomic studies across different exercise modalities, ARK2N was found to be phosphorylated at serine 67 (S67) by AMP-activated protein kinase (AMPK) during various physical activities, including endurance, sprint, and resistance exercises [42]. Phosphorylation of ARK2N at S67 appears to enhance muscle contractile force, an effect supported by in vivo studies where phospho-mimetic mutations (S66/67D) in mice restored skeletal muscle function impaired by *Ark2n* knockout [43].

ARK2N localizes within both the nucleus and contractile apparatus of skeletal muscle fibers, with a preference for fast-twitch fibers that are essential for high-power, rapid contractions [43]. Through affinity purification and mass spectrometry, a diverse ARK2N interactome was identified, including proteins involved in nucleocytoplasmic transport, GTPase signaling, and the cytoskeletal contractile machinery. This interactome was particularly enriched for interactions dependent on AMPK activity and S67 phosphorylation, suggesting that ARK2N functions as a scaffold or adapter in muscle-specific signaling complexes. A key phenotype observed in *Ark2n*-knockout (KO) mice is impaired calcium handling within fast-twitch muscle fibers. Calcium handling in the sarcoplasmic reticulum (SR) is essential for muscle contraction, and *Ark2n*-KO mice exhibit reduced SR calcium loading and increased passive calcium leak, resulting in decreased muscle force production. This defect was not observed in slow-twitch fibers, highlighting ARK2N’s specific role in muscle fiber types associated with rapid, forceful contractions. KO mice lacking *Ark2n* show reduced endurance and performance in forced treadmill running tests, underscoring the gene’s contribution to physical performance under high muscular demand [43]. This phenotype suggests that *ARK2N* not only regulates muscle force at the cellular level but also impacts whole-body exercise tolerance and energy metabolism.

Calcium ions (Ca^2^⁺) play an essential role in muscle contraction and relaxation, which are fundamental to generating strength. When a muscle cell receives a signal to contract, calcium is released from the SR into the cytoplasm. This increase in cytoplasmic calcium binds to troponin, initiating a cascade that allows actin and myosin—the muscle’s contractile proteins—to interact and produce force. This process relies heavily on effective calcium transport systems, including release and reuptake via the SR, regulated by the sarcoplasmic/endoplasmic reticulum Ca^2^⁺-ATPase (SERCA) pump [52]. Proper functioning of these systems ensures efficient contraction and relaxation cycles, which are necessary for optimal strength production. Research highlights that variations in calcium handling directly influence muscle strength. For example, dysfunctions in calcium release or uptake, often seen with age or fatigue, can reduce force generation capacity [53]. In fast-twitch muscle fibers, which are geared toward power and strength, calcium dynamics are particularly critical as they allow for rapid, forceful contractions. Disruptions in calcium signaling, whether from aging or metabolic disturbances, can impair muscle strength by affecting calcium sensitivity in the contractile machinery, thereby reducing force production [54]. This understanding supports our findings on the preferential expression of ARK2N in power athletes and its association with type IIA muscle fiber composition. The observed larger CSA among T allele carriers may relate to enhanced calcium dynamics and contractile efficiency mediated by ARK2N, which are likely advantageous for the maximal strength and rapid contractions required in competitive sports. 

The present study has several limitations that should be acknowledged. First, the sample size of Turkish elite athletes was limited due to the rarity of such athletes, which may have affected the study’s statistical power and the generalizability of the findings. Future studies should aim to include larger cohorts to improve statistical reliability and enhance the robustness of the results. Second, this study focused solely on a single polymorphism of the *ARK2N* gene, potentially overlooking the contributions of other genetic variants or polygenic interactions that may influence muscle fiber CSA and strength performance. Future research could incorporate genome-wide association studies (GWAS) or analyses of additional candidate genes to provide a more comprehensive understanding. Third, the cross-sectional design of this study limits the ability to infer causality between the *ARK2N* variant and muscle fiber CSA. Longitudinal studies tracking genetic and phenotypic changes over time would help establish causal relationships. Fourth, personal best (PB) performances were used as a proxy for athletic performance, which may not fully capture the dynamic and multifaceted nature of strength-related capabilities. Incorporating standardized performance tests and training data in future studies could provide a more holistic assessment. Finally, this study focused on a specific population of Turkish athletes, which limits the generalizability of the findings to other populations with diverse geographic and genetic ancestries. Expanding the research to include athletes from different backgrounds and conducting meta-analyses, as seen in robust studies [55,56,57,58,59], would help to validate the findings and identify population-specific effects. Additionally, the absence of epigenetic analyses in this study represents a significant limitation, as environmental and regulatory factors likely play a role in modulating the genetic effects observed [60,61]. Future studies should incorporate epigenetic profiling to explore gene–environment interactions comprehensively.

On the other hand, the strengths of this study include the use of multiple cohorts and traits to support the hypothesis, along with a focus on world-class athletes to validate the findings. Previous studies investigating genetic markers associated with athletic performance, such as *ACTN3* and *ACE*, have primarily relied on case-control study designs to explore the relationship between genetic variants and strength-athlete status. While these studies have provided valuable insights, they often lack functional data to support the observed associations. In contrast, our study adopts a more integrative approach by combining gene-expression data and the demonstrated link between the *ARK2N* genetic variant and muscle fiber cross-sectional area. This dual approach not only establishes a robust association between the *ARK2N* gene and strength-athlete status but also provides mechanistic insights into how this gene may influence muscle function. Our findings not only highlight the potential role of *ARK2N* in oxidative fast-twitch muscle fibers but also underscore its significance as a novel genetic marker for strength-oriented athletes. By integrating functional and association data, this study builds upon the foundations laid by previous research and sets a precedent for future investigations into the genetic basis of athletic performance.

## 5. Conclusions

In conclusion, our study identifies *ARK2N* as a gene specific to oxidative fast-twitch muscle fibers, with its functional variant associated with increased muscle fiber size and strength-athlete status. These findings have important clinical implications, particularly for conditions like sarcopenia, where muscle mass and strength decline with age. Targeting *ARK2N* could pave the way for novel therapeutic strategies to preserve or enhance muscle function in aging populations and individuals at risk of muscle degeneration. Future research should focus on elucidating the molecular mechanisms of *ARK2N* in muscle adaptation, validating its effects across diverse populations, and investigating its potential as a therapeutic target for sarcopenia and other muscle-related conditions.

## Figures and Tables

**Table 1:** Genotype and allele frequencies of the Arkadia (RNF111) N-terminal-like PKA signaling regulator 2N (*ARK2N*) rs6507691 polymorphism in athletes and controls.

Group	*ARK2N* Genotypes			T Allele Frequency, %	*p*-Value
	TT	CT	CC		
*Turkish cohorts*					
Power athletes (*n* = 31)	4	13	14	33.9	NS
Endurance athletes (*n* = 29)	4	12	13	34.5	NS
Controls #1 (*n* = 20)	2	11	7	37.5	–
Controls #2 (*n* = 557)	N/A	N/A	N/A	29.8	–
*Russian cohorts*					
World-class strength athletes (*n* = 22)	2	13	7	38.6	0.023 *
Elite strength athletes (*n* = 68)	5	23	40	24.3	0.631
World-class wrestlers (*n* = 34)	4	15	15	33.8	0.044 *
Elite wrestlers (*n* = 91)	11	36	44	31.9	0.016 *
All Russian athletes (*n* = 215)	22	87	106	30.5	0.0078 *
Russian controls (*n* = 182)	9	62	111	22.0	–

* *p* < 0.05, statistically significant differences in allelic frequencies between athletic cohorts and ethnically matched control groups. N/A, not available; NS, non-significant (compared to both Turkish control groups).

## Data Availability

The data presented in this study are available on request from the corresponding author.

## Supplementary Materials

The following supporting information can be downloaded at: https://www.mdpi.com/article/10.3390/metabo14120684/s1, Table S1: Relationship between the *ARK2N* rs6507691 polymorphism and competitive performance among power athletes; Table S2: Relationship between the *ARK2N* rs6507691 polymorphism and competitive performance among endurance athletes; Table S3: Relationship between the *ARK2N* rs6507691 polymorphism and competitive performance among all athletes.
