# Supplementary material for: The ARK2N (C18ORF25) Genetic Variant Is Associated with Muscle Fiber Size and Strength Athlete Status
Source: Metabolites. 2024 Dec 5;14(12):684. doi: 10.3390/metabo14120684 (PMC11676174; doi:10.3390/metabo14120684)
Supplement: Supplementary file 1 [file metabolites-14-00684-s001.zip › metabolites-3340042-supplementary.pdf]

Supplementary file

Table S1. Relationship between the *ARK2N* rs6507691 polymorphism and competitive performance among Turkish power athletes.

Model	Genotype	n	Mean score (PB)	Difference (95% CI)	P-value
Co-dominant	CC	14	962.07	0.00	0.72
	TC	13	1007.38	30.94 (-50-54 to 112.41)	
	TT	4	1007.75	33.89 (-80.87 to 148.66)	
Dominant	CC	14	962.07	0.00	0.42
	TC-TT	17	1007.47	31.67 (-43.44 to 106.78)	
Recessive	CC-TC	27	983.89	0.00	0.74
	TT	4	1007.75	17.73 (-87.96 to 123.42)	
Over-dominant	CC-TT	18	972.22	0.00	0.57
	TC	13	1007.38	22.01 (-52.72 to 96.74)	

Table S2. Relationship between the *ARK2N* rs6507691 polymorphism and competitive performance among Turkish endurance athletes.

Model	Genotype	n	Mean score (PB)	Difference (95% CI)	P-value
Co-dominant	CC	13	1024.46	0.00	0.46
	TC	12	1013.25	-15.98 (-73.69 to 41.73)	
	TT	4	1041.25	40.73 (-47.21 to 128.67)	
Dominant	CC	13	1024.46	0.00	0.9
	TC-TT	16	1020.25	-3.65 (-58.70 to 51.41)	
Recessive	CC-TC	25	1019.08	0.00	0.26
	TT	4	1041.25	48.58 (-33.49 to 130.64)	
Over-dominant	CC-TT	17	1028.41	0.00	0.38
	TC	12	1013.25	-24.59 (-79.03 to 29.84)	

Table S3. Relationship between the *ARK2N* rs6507691 polymorphism and competitive performance among all Turkish athletes.

Model	Genotype	n	Mean score (PB)	Difference (95% CI)	P-value
Co-dominant	CC	27	992.11	0.00	0.72
	TC	25	1010.2	14.33 (-35.86 to 64.51)	
	TT	8	1024.5	28.76 (-45.53 to 103.05)	
Dominant	CC	14	962.07	0.00	0.47
	TC-TT	17	1007.47	17.48 (-29.75 to 64.70)	
Recessive	CC-TC	27	983.89	0.00	0.55
	TT	4	1007.75	21.05 (-47.72 to 89.81)	
Over-dominant	CC-TT	18	972.22	0.00	0.76
	TC	13	1007.38	7.26 (-39.31 to 53.82)	
